# Preoperative Systemic Inflammatory Indices as Adjunctive Biomarkers for Malignancy Prediction in Bethesda III (AUS) Thyroid Nodules

**DOI:** 10.3390/cancers18193170

**Published:** 2026-10-01

**Authors:** Gökhan Rıza Baykal, Müge Keskin, Kübra Katipoğlu, Ersin Gürkan Dumlu, Didem Özdemir, Oya Topaloğlu, Reyhan Ersoy, Bekir Çakır

**Affiliations:** 1 Department of Endocrinology and Metabolism, Ankara Bilkent City Hospital, Ankara 06800, Türkiye; 2 Department of Endocrinology and Metabolism, Ankara Bilkent City Hospital, Health Sciences University, Ankara 06800, Türkiye; 3 Department of Pathology, Ankara Bilkent City Hospital, Ankara 06800, Türkiye; 4 Department of General Surgery, Faculty of Medicine, Ankara Yildirim Beyazit University, Ankara 06800, Türkiye; 5 Department of Endocrinology and Metabolism, Faculty of Medicine, Ankara Yildirim Beyazit University, Ankara 06800, Türkiye

**Keywords:** atypia of undetermined significance, thyroid cancer, thyroid nodules, systemic immune-inflammation index, malignancy prediction

## Abstract

**Background/Objectives:** Atypia of undetermined significance (AUS; Bethesda category III) remains a challenging thyroid cytological diagnosis because of its heterogeneous malignancy risk and limited preoperative certainty. Molecular testing can improve risk stratification but is not universally available. This study evaluated the diagnostic and incremental predictive value of preoperative complete blood count-derived systemic inflammatory indices in surgically treated Bethesda III thyroid nodules. **Methods:** This retrospective cohort included 352 patients who underwent thyroidectomy for Bethesda III thyroid nodules. Preoperative neutrophil-to-lymphocyte ratio (NLR), platelet-to-lymphocyte ratio (PLR), lymphocyte-to-monocyte ratio (LMR), systemic immune-inflammation index (SII), systemic inflammation response index (SIRI), and aggregate index of systemic inflammation (AISI) were evaluated. Postoperative histopathology served as the reference standard. Receiver operating characteristic analysis and multivariable logistic regression were performed, followed by incremental model assessment, bootstrap internal validation, Hashimoto thyroiditis interaction analysis, and exploratory molecular subgroup analysis. **Results:** Among 352 patients, 197 (56.0%) had malignant histopathology. Malignant nodules showed higher NLR, PLR, SII, SIRI, and AISI and lower LMR. All six indices remained independently associated with malignancy after multivariable adjustment, with the strongest positive associations observed for SII (adjusted OR, 2.09; 95% CI, 1.60–2.72) and NLR (adjusted OR, 1.96; 95% CI, 1.51–2.54). SII demonstrated the numerically highest individual discrimination (AUC 0.687; 95% CI, 0.631–0.740), followed by NLR (AUC 0.661) and AISI (AUC 0.659); SII and NLR did not differ significantly (DeLong *p* = 0.182). Addition of SII increased the AUC of the clinico-ultrasonographic model from 0.695 to 0.761, with an optimism-corrected AUC of 0.721. Hashimoto thyroiditis did not significantly modify these associations. In the molecular subgroup, mutation status increased model discrimination from 0.765 to 0.824, whereas no statistically significant additional improvement with SII was detected in the exploratory molecular subgroup. **Conclusions:** Preoperative systemic inflammatory indices, particularly SII, provide modest but incremental predictive information beyond conventional clinical and ultrasonographic variables in surgically treated Bethesda III thyroid nodules. Their role appears complementary rather than diagnostic when used in isolation, particularly when comprehensive molecular testing is unavailable. Prospective external validation is required before clinical implementation.

## Simple Summary

Atypia of undetermined significance (AUS; Bethesda category III) is a common but challenging thyroid cytology diagnosis because the risk of cancer varies widely among patients. Molecular testing can improve risk assessment, but it is not universally available and may be costly. We investigated whether simple inflammation-related markers calculated from routine blood counts could help improve malignancy prediction in patients with AUS thyroid nodules. In 352 surgically treated patients, several inflammatory indices were associated with malignant histopathology. The systemic immune-inflammation index (SII) showed the highest numerical performance among the evaluated markers and improved prediction when added to clinical and ultrasound findings. However, its diagnostic performance alone was only modest, and it did not provide additional value when molecular information was already available. These findings suggest that SII may add useful, low-cost information to conventional clinical and ultrasonographic risk assessment.

## 1. Introduction

Thyroid nodules are among the most common endocrine disorders encountered in clinical practice, with high-resolution ultrasonography detecting nodules in up to 60–70% of adults [1,2]. Although the vast majority of thyroid nodules are benign, approximately 5–15% are malignant, making accurate preoperative risk stratification essential to avoid both unnecessary surgery and delayed cancer treatment. Ultrasound-guided fine-needle aspiration biopsy (FNAB) remains the cornerstone of thyroid nodule evaluation and is routinely reported according to the Bethesda System for Reporting Thyroid Cytopathology [3,4,5].

Among the Bethesda categories, atypia of undetermined significance (AUS; Bethesda category III) represents one of the greatest diagnostic challenges in thyroid cytopathology. According to the 2023 Bethesda System for Reporting Thyroid Cytopathology, AUS encompasses specimens showing limited cytological atypia that is insufficient for a definitive benign or malignant diagnosis [5]. Reported malignancy rates in AUS vary substantially across institutions and study cohorts, particularly in surgical series, reflecting differences in patient selection, cytological interpretation, ultrasound findings, molecular testing, and thresholds for thyroidectomy [6,7,8]. Consequently, the optimal management of AUS nodules remains controversial and requires individualized decision-making based on clinical, ultrasonographic, cytological, and, when available, molecular findings.

Over the past decade, molecular testing has substantially improved the diagnostic evaluation of indeterminate thyroid nodules. Identification of oncogenic alterations, including BRAF, RAS, RET/PTC, and other driver mutations, has enabled more accurate estimation of malignancy risk and has influenced surgical planning [9,10]. Nevertheless, molecular analyses remain costly, are not universally available, and are not routinely performed in many healthcare systems because of financial and logistical limitations [11]. Therefore, inexpensive, readily available, and reproducible biomarkers that can complement conventional diagnostic methods remain highly desirable.

Chronic inflammation has increasingly been recognized as a key contributor to thyroid carcinogenesis. Experimental and clinical studies have demonstrated that inflammatory signaling promotes oxidative stress, cytokine-mediated cellular proliferation, angiogenesis, immune evasion, and remodeling of the tumor microenvironment, thereby facilitating tumor initiation and progression [12,13]. In addition to local inflammatory processes, thyroid malignancy is associated with systemic inflammatory and immune changes that may be reflected in peripheral blood cell indices [14].

Systemic immune-inflammatory biomarkers derived from routine complete blood counts have emerged as readily available indicators of cancer-associated inflammation. These include the neutrophil-to-lymphocyte ratio (NLR), platelet-to-lymphocyte ratio (PLR), lymphocyte-to-monocyte ratio (LMR), systemic immune-inflammation index (SII), systemic inflammation response index (SIRI), and aggregate index of systemic inflammation (AISI). Unlike single hematological parameters, these composite indices integrate multiple circulating inflammatory cell populations and provide a more comprehensive assessment of the balance between protumor inflammatory activity and host antitumor immune surveillance. Elevated CBC-derived inflammatory indices have been associated with adverse clinicopathological characteristics and poorer clinical outcomes across a wide range of solid malignancies [15,16,17]. In thyroid cancer, however, the available evidence remains heterogeneous, and the clinical utility of these biomarkers for diagnostic and prognostic purposes requires further prospective validation [18,19]. In papillary thyroid carcinoma, elevated preoperative NLR and SII have been associated with adverse clinicopathological features, particularly lymph node metastasis, although associations with other pathological characteristics have been inconsistent across studies [20,21]. CBC-derived inflammatory indices have also shown potential to distinguish benign from malignant thyroid nodules; however, current evidence does not support their routine use as validated risk-prediction tools [22].

Evidence specifically addressing AUS nodules remains limited and is largely derived from small, single-center retrospective studies. Available studies have evaluated several CBC-derived inflammatory indices, including NLR, PLR, LMR, SII, and SIRI, but findings have been heterogeneous, with no single marker demonstrating consistently robust independent predictive value across cohorts [23,24,25].

Consequently, comprehensive studies evaluating multiple systemic inflammatory indices in large, homogeneous cohorts of surgically treated AUS nodules while accounting for important confounding factors, such as chronic autoimmune thyroiditis, and incorporating established risk stratification variables, including ultrasonographic and molecular findings, remain scarce. Although ultrasonographic risk-stratification systems and molecular testing have improved the evaluation of indeterminate thyroid nodules, they do not eliminate the diagnostic uncertainty associated with AUS cytology. Ultrasonographic features can further stratify malignancy risk within AUS nodules, but performance differs across cytologic subgroups; molecular classifiers provide useful rule-out or rule-in information, yet their diagnostic performance is not absolute and depends on the tested population, disease prevalence, and the molecular platform used [10,26].

Access to molecular testing and its use also vary across healthcare systems because of cost, reimbursement policies, availability of specialized molecular laboratories, and logistical barriers [11]. Consequently, readily obtainable blood-based inflammatory biomarkers may provide complementary diagnostic information with minimal additional cost or workflow burden. Identifying reliable biomarkers capable of refining malignancy risk assessment in this diagnostically challenging subgroup may facilitate more individualized patient management.

Therefore, the present study aimed to comprehensively evaluate six preoperative systemic immune-inflammatory indices, NLR, PLR, LMR, SII, SIRI, and AISI, in a relatively large cohort of patients undergoing thyroidectomy for AUS thyroid nodules. We investigated their association with postoperative histopathological outcomes, assessed their diagnostic performance for predicting malignancy, and evaluated whether these associations were independent of coexisting chronic autoimmune thyroiditis. We further explored their potential complementary value alongside conventional ultrasonographic and molecular approaches to improve preoperative malignancy risk stratification.

## 2. Materials and Methods

### 2.1. Study Design and Patient Selection

This retrospective observational cohort study was conducted at the Department of Endocrinology and Metabolism, Ankara Bilkent City Hospital, a high-volume tertiary referral center for thyroid diseases. Consecutive adult patients who underwent thyroidectomy for thyroid nodules with a preoperative cytological diagnosis of AUS between January 2019 and July 2025 were identified through the institutional electronic medical record system, cytopathology database, operative records, and pathology archives.

Clinical, laboratory, ultrasonographic, cytological, molecular, operative, and histopathological data were extracted using a standardized electronic case report form. Only patients with definitive postoperative histopathological confirmation were included in the final analysis. The relatively low proportion of missing data was facilitated by the availability of routinely collected preoperative laboratory and surgical data and by cross-referencing multiple institutional data sources. Because the study population consisted exclusively of surgically treated patients, definitive postoperative histopathology and routine preoperative complete blood count measurements were available for nearly all potentially eligible cases.

For patients with multiple thyroid nodules, analyses were based on the index nodule, defined as the nodule that underwent ultrasound-guided FNAB and was classified as AUS before surgery. When more than one AUS nodule was present, the biopsied nodule corresponding to the surgical pathology specimen was considered the index lesion.

Eligible patients were required to be 18 years of age or older, have available preoperative complete blood count (CBC) measurements obtained within 30 days before thyroidectomy, and have complete clinicopathological data necessary for calculation of systemic inflammatory indices. Patients were excluded if they had active infection or acute inflammatory disease at the time of blood sampling, hematological disorders affecting peripheral blood cell counts, concurrent non-thyroid malignancies, systemic corticosteroid or immunosuppressive therapy at the time of blood sampling, or incomplete clinical or pathological data. Patients with chronic autoimmune thyroiditis (Hashimoto thyroiditis) were not excluded because this condition was prespecified as an important potential confounder and was evaluated in predefined subgroup analyses.

The primary outcome was the final postoperative histopathological diagnosis of the aspirated index nodule, categorized as benign or malignant. The index nodule was matched to the corresponding lesion in the surgical pathology report. Malignancy was defined according to the histopathological diagnosis of the aspirated AUS index nodule; incidental carcinomas arising exclusively in separate non-index nodules were not considered malignant outcomes for the primary analysis. Malignant lesions consisted of papillary thyroid carcinoma, including its histological variants. Benign lesions included nodular hyperplasia, follicular adenoma, non-invasive follicular thyroid neoplasm with papillary-like nuclear features (NIFTP), Hashimoto thyroiditis without coexisting carcinoma, and other benign thyroid lesions. Indications for thyroidectomy reflected routine multidisciplinary clinical practice and included cytological, ultrasonographic, clinical, patient preference, and molecular considerations where available. The present study was designed to evaluate factors associated with postoperative malignancy within surgically treated Bethesda III nodules rather than determinants of surgical selection. Detailed study-level clinical and ultrasonographic data were collected for patients who proceeded to surgery, whereas corresponding structured data were not available for patients managed without surgery.

**Figure 1:**
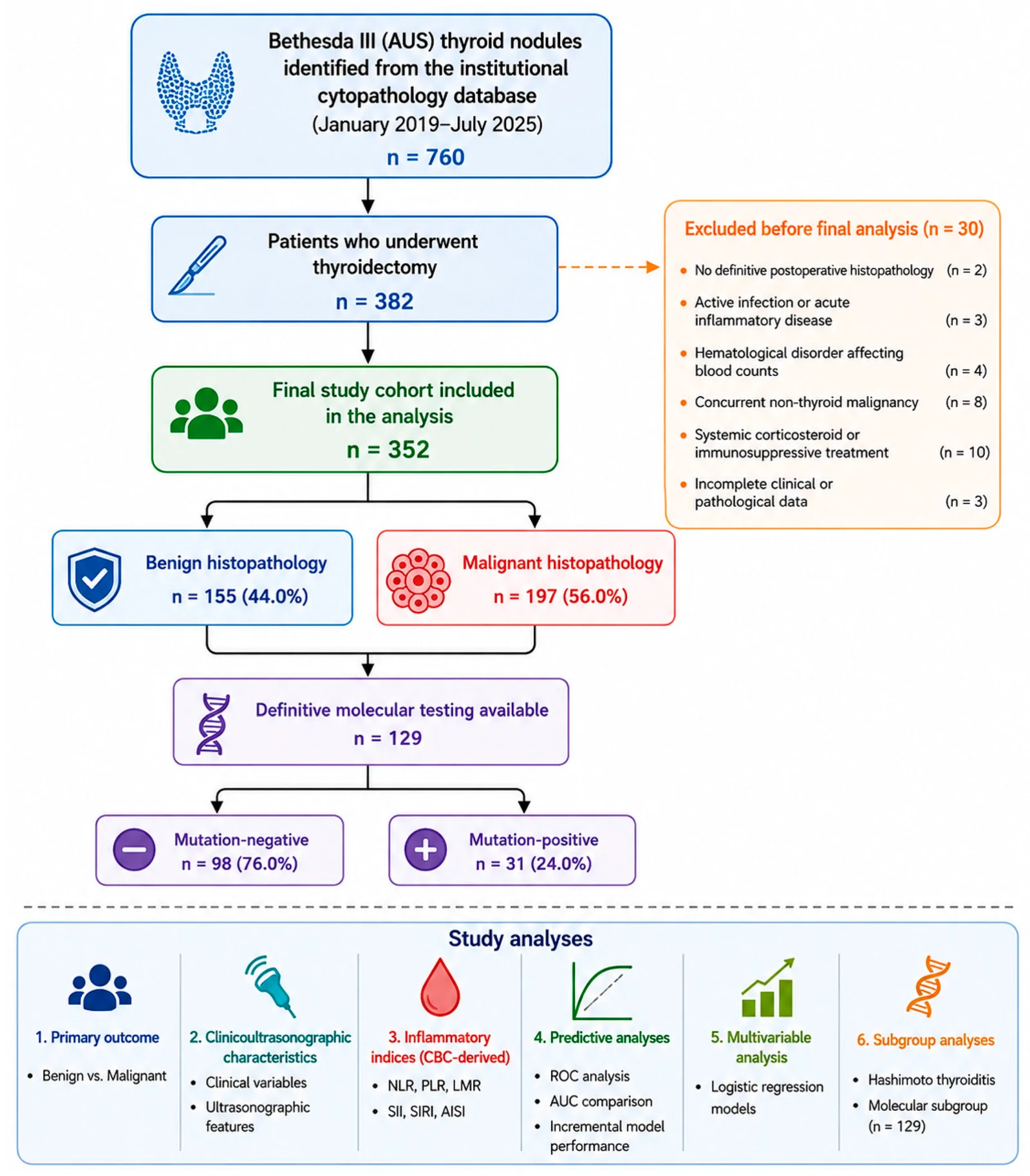
Flow diagram of patient selection and study design. AUS, atypia of undetermined significance; CBC, complete blood count; NLR, neutrophil-to-lymphocyte ratio; PLR, platelet-to-lymphocyte ratio; LMR, lymphocyte-to-monocyte ratio; SII, systemic immune-inflammation index; SIRI, systemic inflammation response index; AISI, aggregate index of systemic inflammation.

A flow diagram illustrating patient selection, exclusion criteria, and the final study cohort is presented in Figure 1.

### 2.2. Clinical, Laboratory, Cytological and Histopathological Data Collection

Clinical, laboratory, cytological, molecular, surgical, and histopathological data were retrospectively retrieved from the institutional electronic medical record system using a predefined standardized data collection form. Demographic variables included age and sex. Recorded metabolic comorbidities included hypertension, diabetes mellitus, hyperlipidemia, and obesity, which were additionally evaluated as potential confounders of systemic inflammatory indices. Obesity was defined as a body mass index (BMI) ≥30 kg/m^2^. Laboratory variables comprised thyroid-stimulating hormone (TSH), free thyroxine (fT4), anti-thyroid peroxidase antibody (anti-TPO), anti-thyroglobulin antibody (anti-Tg), and CBC parameters.

Chronic autoimmune thyroiditis (Hashimoto thyroiditis) was defined according to the presence of characteristic ultrasonographic findings and/or positive thyroid autoantibodies. Because the upper reference limit for anti-thyroglobulin antibody changed during the study period following modification of the laboratory assay, anti-Tg positivity was determined according to the assay-specific reference range in use at the time of measurement. Similarly, anti-TPO positivity was defined using the corresponding assay-specific upper reference limit. Among the 39 patients classified as having Hashimoto thyroiditis, all had characteristic heterogeneous thyroid parenchyma on ultrasonography. Thirty-six patients (92.3%) also had positive thyroid autoantibodies, whereas three patients (7.7%) fulfilled the ultrasonographic criterion alone. No patient was classified on the basis of serological findings alone. Histological evidence of chronic lymphocytic thyroiditis was not systematically captured in the analytic dataset and was therefore not incorporated into the prespecified definition.

Cytological diagnoses were retrieved from the original pathology reports, and only nodules classified as AUS were included. Cytological terminology was aligned with the 2023 Bethesda System for Reporting Thyroid Cytopathology [5]. Histopathological diagnoses were based on the final surgical pathology reports and classified as benign or malignant in accordance with the 2022 World Health Organization Classification of Thyroid Neoplasms [27]. NIFTP was classified as non-malignant for the primary binary outcome. Histological subtype, tumor size, multifocality, and other available pathological characteristics were recorded for malignant lesions. However, a standardized Bethesda III subclassification (nuclear atypia vs. other patterns of atypia) was not consistently documented in the original cytology reports throughout the study period and therefore could not be reliably incorporated into the multivariable models. When available, molecular testing results were retrieved from the institutional molecular pathology database. The presence of BRAF, KRAS, and NRAS mutations was recorded for molecular subgroup analyses.

### 2.3. Ultrasonographic Evaluation

Preoperative ultrasonographic examinations and ultrasound-guided FNABs were performed by experienced endocrinologists dedicated to the thyroid clinic using high-resolution ultrasound systems. In patients with multiple thyroid nodules, only the index nodule, defined as the nodule that underwent FNAB and received a cytological diagnosis of AUS, was included in the ultrasonographic analyses.

Ultrasonographic characteristics were retrospectively extracted from the original ultrasound reports using a predefined standardized data collection form. The variables recorded for the index nodule included maximum nodule diameter, anatomical location, composition, echogenicity, margin characteristics, and calcification pattern.

Anatomical location was categorized as right lobe, left lobe, or isthmus. Nodule composition was classified as solid, predominantly solid, or predominantly cystic. Echogenicity was categorized as isoechoic, hypoechoic, or iso-/hypoechoic according to the original ultrasonographic description. Margins were classified as smooth, irregular, or ill-defined. Calcification pattern was categorized as absent, macrocalcification, or microcalcification. These ultrasonographic variables were evaluated for their association with postoperative histopathological diagnosis and were included, where applicable, in the clinico-ultrasonographic multivariable models. Because the examinations were retrospectively extracted from routine clinical reports, a standardized TI-RADS category could not be reconstructed reliably for all patients, as all required TI-RADS components were not consistently documented throughout the study period.

### 2.4. Molecular Analysis

Beginning in 2024, molecular testing was incorporated into the routine evaluation of selected patients with AUS thyroid nodules at our institution. Molecular analyses were performed on ultrasound-guided FNAB specimens when additional preoperative diagnostic information was considered clinically beneficial during multidisciplinary decision-making. Available molecular data were retrospectively retrieved from the institutional molecular pathology database.

The molecular panel included analysis of the BRAF V600E mutation and RAS hotspot mutations, comprising KRAS and NRAS codon 61 (Q61H, Q61K, Q61L, and Q61R) variants. Patients were classified as mutation-positive if at least one pathogenic mutation was detected and mutation-negative when none of the tested mutations were identified. Individual mutation frequencies were also recorded for descriptive analyses.

Because molecular testing was introduced during the latter part of the study period and was not routinely performed in all patients, analyses involving molecular variables were restricted to the subgroup with available genetic results. Molecular findings were evaluated both independently and in combination with ultrasonographic characteristics and systemic inflammatory indices to explore their incremental value in predicting postoperative malignancy.

### 2.5. Laboratory Measurements and Calculation of Systemic Inflammatory Indices

Preoperative laboratory data were retrieved from the institutional electronic medical record system. Complete blood count parameters obtained within 30 days before thyroidectomy were included in the analysis. When more than one measurement was available during this period, the value closest to the date of surgery was used. Measurements were obtained from routine preoperative blood samples analyzed in the hospital laboratory. Total leukocyte, absolute neutrophil, lymphocyte, and monocyte counts were expressed as ×10^9^/L, whereas platelet counts were expressed as ×10^9^/L.

Patients with evidence of active infection or an acute inflammatory condition at the time of blood sampling were excluded. Patients with hematological disorders that could alter peripheral blood cell counts, concurrent non-thyroid malignancy, or current systemic corticosteroid or immunosuppressive treatment were also excluded, as described above. Chronic autoimmune thyroiditis was not considered an exclusion criterion and was evaluated as a prespecified potential confounder.

Systemic inflammatory indices were calculated from absolute peripheral blood cell counts using the following formulas:

- Neutrophil-to-lymphocyte ratio (NLR): neutrophil count/lymphocyte count
- Platelet-to-lymphocyte ratio (PLR): platelet count/lymphocyte count
- Lymphocyte-to-monocyte ratio (LMR): lymphocyte count/monocyte count
- Systemic immune-inflammation index (SII): platelet count × neutrophil count/lymphocyte count
- Systemic inflammation response index (SIRI): neutrophil count × monocyte count/lymphocyte count
- Aggregate index of systemic inflammation (AISI): neutrophil count × monocyte count × platelet count/lymphocyte count

All indices were calculated using the laboratory values obtained from the same blood sample. The calculated indices were analyzed both as continuous variables and, where appropriate, as categorical variables using optimal cutoff values derived from receiver operating characteristic curve analyses.

### 2.6. Statistical Analysis

Continuous variables were assessed for distributional characteristics using histograms, quantile–quantile plots, and measures of skewness. Normally distributed variables were summarized as mean ± standard deviation and compared using Student’s *t* test, whereas non-normally distributed variables were presented as median and interquartile range and compared using the Mann–Whitney *U* test. Categorical variables were expressed as numbers and percentages and compared using Pearson’s chi-square test or Fisher’s exact test, as appropriate. For the 2 × 2 comparisons of sex and Hashimoto thyroiditis presented in Table 1, Yates continuity correction was applied. No imputation was performed for missing data. Variables included in the primary multivariable analyses were complete in the final analytic cohort, whereas ancillary analyses were based on available observations.

The primary outcome was postoperative malignant histopathology. Associations between each systemic inflammatory index and malignancy were initially evaluated using univariable logistic regression. Because the inflammatory indices showed right-skewed distributions, all six indices were natural-log transformed and subsequently standardized. Odds ratios (ORs) with 95% confidence intervals (CIs) were therefore expressed per 1-standard-deviation increase in the log-transformed index. Thus, the reported ORs reflect the change in the odds of malignancy per 1-SD increase in the transformed distribution rather than per one-unit increase in the original or logarithmic scale.

Because the inflammatory indices are derived from overlapping hematological components and are therefore substantially correlated, they were not entered simultaneously into the same multivariable model. Instead, each index was evaluated separately in an otherwise identical multivariable logistic regression model adjusted for age, sex, serum thyroid-stimulating hormone level, Hashimoto thyroiditis, maximum index-nodule diameter, nodule composition, echogenicity, margin characteristics, and calcification pattern. Adjusted ORs with 95% CIs were reported. Covariates were selected a priori on the basis of established or clinically plausible relevance to malignancy risk and were not selected using automated stepwise procedures or univariable significance testing. After accounting for the degrees of freedom required for categorical variables, each primary model contained 14 non-intercept parameters, including the inflammatory index. With 197 malignant outcomes, this corresponded to approximately 14 outcome events per model parameter. Multicollinearity among predictors included in the multivariable models was assessed using variance inflation factors (VIFs) after appropriate indicator coding of categorical variables.

The discriminatory performance of individual inflammatory indices was evaluated using receiver operating characteristic (ROC) curve analysis and the area under the curve (AUC) with 95% CIs. AUCs were compared using the DeLong method. Exploratory optimal cutoff values were determined using the maximum Youden index, and corresponding sensitivity and specificity values were calculated. Because these cutoffs were derived and evaluated within the same cohort, cutoff-based analyses were considered exploratory. To provide complementary clinically interpretable estimates, additional multivariable logistic regression analyses were performed after categorizing each inflammatory index according to its ROC-derived optimal cutoff. These analyses used the same covariate adjustment as the primary models and were considered exploratory because the thresholds were derived from the same cohort.

To assess whether systemic inflammatory indices provided predictive information beyond conventional clinical and ultrasonographic variables, a clinico-ultrasonographic base model was constructed using age, sex, serum TSH, Hashimoto thyroiditis, maximum nodule diameter, composition, echogenicity, margin characteristics, and calcification pattern. Each of the six inflammatory indices (NLR, PLR, LMR, SII, SIRI, and AISI) was then added separately to the base model. Incremental performance was assessed using changes in AUC, likelihood-ratio tests, Akaike information criterion (AIC), and Brier scores. For concise presentation, the three indices with the highest individual discriminatory performance (SII, NLR, and AISI) are presented in the main manuscript, whereas incremental model results for all six indices are provided in Supplementary Table S1.

To examine whether comparable predictive performance could be achieved with a less complex model, an exploratory parsimonious model was additionally evaluated. This model retained age, sex, serum TSH, Hashimoto thyroiditis, nodule composition, and margin characteristics on the basis of clinical relevance and the observed contribution of the ultrasonographic variables, while maximum nodule diameter, echogenicity, and calcification pattern were omitted. Model performance was compared with that of the fully adjusted model using AUC, AIC, Brier score, likelihood-ratio testing, and bootstrap-corrected performance measures.

Model calibration was evaluated using calibration plots and calibration slopes. Internal validation was performed using 2000 bootstrap resamples to estimate optimism-corrected AUCs, Brier scores, and calibration slopes. Decision curve analysis was used to compare the net benefit of the base and inflammatory-index-extended models across threshold probabilities ranging from 0.10 to 0.80.

Prespecified subgroup analyses were performed according to the presence or absence of Hashimoto thyroiditis. Effect modification was evaluated by including an interaction term between each standardized log-transformed inflammatory index and Hashimoto thyroiditis status in multivariable logistic regression models.

As a sensitivity analysis, the multivariable models were additionally adjusted for hypertension, diabetes mellitus, hyperlipidemia, and obesity to assess potential confounding by metabolic conditions associated with chronic low-grade inflammation.

Molecular analyses were restricted to patients with definitive BRAF, KRAS, and NRAS results and were considered exploratory because molecular testing was introduced during the later study period and was not routinely performed in the entire cohort. Within this subgroup, the association between mutation status and malignant histopathology was evaluated, and nested clinico-ultrasonographic, clinico-ultrasonographic-plus-molecular, and clinico-ultrasonographic-plus-molecular-plus-SII models were compared using AUC, likelihood-ratio testing, AIC, and Brier scores.

All statistical tests were two-sided, and a *p* value < 0.05 was considered statistically significant. Statistical analyses were performed using IBM SPSS Statistics, version 30.0 (IBM Corp., Armonk, NY, USA) and Python, version 3.13.5. Logistic regression modeling, bootstrap internal validation, calibration analyses, decision-curve analysis, and graphical visualization were additionally performed in Python using the statsmodels, scikit-learn, SciPy, NumPy, pandas, and Matplotlib (version 3.10.8) packages.

### 2.7. Use of Generative Artificial Intelligence

ChatGPT (GPT-5.6, OpenAI) was used as an AI-assisted writing tool during manuscript preparation to support English-language refinement, improve clarity and readability, and assist with the organization of selected portions of the manuscript. The tool was not used to generate study data, perform statistical analyses, alter or manipulate results, or independently determine the scientific conclusions of the study. All AI-assisted output was critically reviewed, edited, and verified by the authors.

## 3. Results

### 3.1. Study Population

A total of 352 patients with surgically treated Bethesda category III thyroid nodules and definitive postoperative histopathological diagnoses were included in the final analysis. Of these, 155 patients (44.0%) had benign histopathology and 197 patients (56.0%) had malignant histopathology. In all 197 malignant cases, malignancy was confirmed in the aspirated AUS index nodule rather than representing an incidental carcinoma arising exclusively in another thyroid nodule. All malignant index nodules were papillary thyroid carcinomas; no non-papillary thyroid carcinomas were identified. Of the 197 malignant index nodules, 66 (33.5%) measured ≤10 mm and were classified as papillary thyroid microcarcinomas.

### 3.2. Baseline Characteristics

The baseline clinical, laboratory, and ultrasonographic characteristics of the study population are presented in Table 1. The median age was 52 years in the benign group and 50 years in the malignant group, with no significant difference between the groups (*p* = 0.321). Sex distribution was also similar between the benign and malignant groups (*p* = 0.070). The prevalence of hypertension, diabetes mellitus, hyperlipidemia, and obesity did not differ significantly between the benign and malignant groups (all *p* > 0.05).

Serum TSH levels were significantly higher in patients with malignant histopathology than in those with benign histopathology (median, 1.60 vs. 1.21 mIU/L; *p* = 0.039). In contrast, free thyroxine levels and maximum nodule diameter did not differ significantly between the groups.

Hashimoto thyroiditis was significantly more frequent among patients with benign histopathology than among those with malignant histopathology (15.5% vs. 7.6%; *p* = 0.030). Nodule composition also differed significantly according to histopathological outcome (*p* = 0.019), with solid nodules occurring more frequently in the malignant group (43.7% vs. 34.2%) and predominantly cystic nodules occurring more frequently in the benign group (8.4% vs. 2.5%).

Margin characteristics showed the strongest ultrasonographic association with malignancy (*p* < 0.001). Irregular margins were observed in 14.2% of malignant nodules compared with 3.9% of benign nodules, while ill-defined margins were also more frequent in the malignant group (37.6% vs. 25.8%). In contrast, nodule side, echogenicity, and calcification pattern were not significantly different between the groups.

### 3.3. Systemic Inflammatory Indices

Patients with malignant histopathology had significantly higher preoperative NLR, PLR, SII, SIRI, and AISI values than those with benign histopathology. Median NLR was 2.06 in the malignant group and 1.75 in the benign group, while median SII values were 547.3 and 422.3, respectively. PLR, SIRI, and AISI were also significantly elevated in malignant nodules (all *p* < 0.001). Conversely, LMR was significantly lower in the malignant group than in the benign group (5.80 vs. 6.14; *p* = 0.008). Detailed distributions of the inflammatory indices are presented in Table 2.

### 3.4. Diagnostic Performance

**Figure 2:**
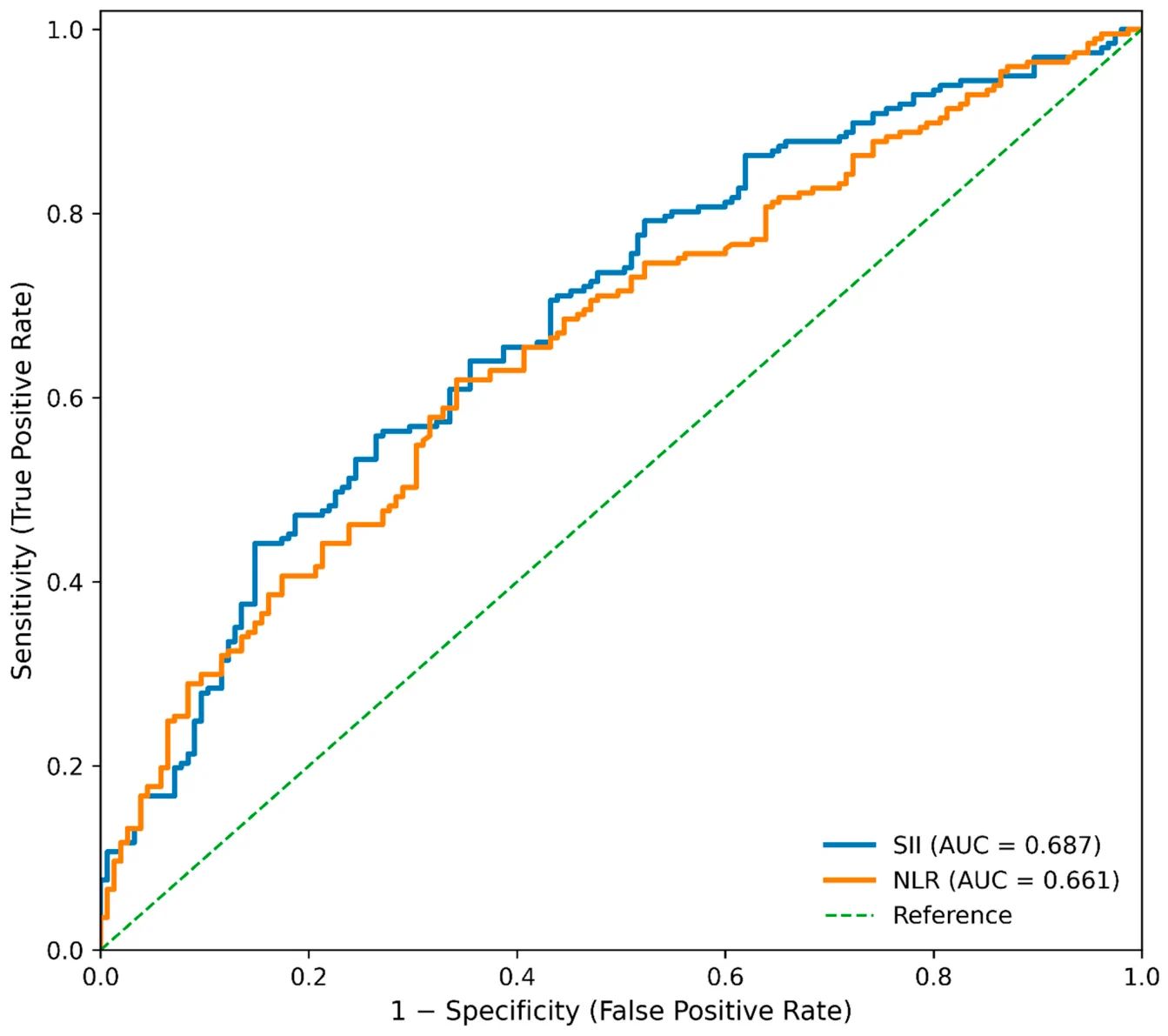
Receiver operating characteristic curves of the systemic immune-inflammation index and neutrophil-to-lymphocyte ratio for predicting malignant histopathology in Bethesda III thyroid nodules. SII demonstrated an AUC of 0.687, whereas NLR demonstrated an AUC of 0.661. The diagonal dashed line represents the reference line of no discrimination. **Abbreviations:** AUC, area under the curve; NLR, neutrophil-to-lymphocyte ratio; SII, systemic immune-inflammation index.

Among the evaluated systemic inflammatory indices, SII demonstrated the numerically highest discriminatory performance for malignant histopathology, with an area under the receiver operating characteristic curve of 0.687 (95% CI, 0.631–0.740). NLR showed the second-highest performance, with an AUC of 0.661 (95% CI, 0.603–0.718), followed closely by AISI with an AUC of 0.659 (95% CI, 0.602–0.714). The corresponding AUC values were 0.634 for SIRI, 0.627 for PLR, and 0.582 for LMR. Although SII showed a higher AUC than NLR, the difference between the two indices was not statistically significant (DeLong *p* = 0.182). Overall, the individual inflammatory indices demonstrated modest discriminatory ability. Detailed diagnostic performance measures are presented in Table 3, and the ROC curves for SII and NLR are shown in Figure 2.

### 3.5. Multivariable Logistic Regression Analysis

Univariable logistic regression analyses were performed to evaluate the association between each systemic inflammatory index and malignant histopathology. Because the inflammatory indices showed right-skewed distributions, they were natural-log transformed and subsequently standardized. Odds ratios were therefore expressed per 1-standard-deviation increase in the log-transformed index.

In univariable analyses, higher NLR, PLR, SII, SIRI, and AISI values were significantly associated with increased odds of malignant histopathology. The strongest association was observed for SII (OR, 2.09; 95% CI, 1.63–2.69; *p* < 0.001). Higher NLR (OR, 1.84; 95% CI, 1.45–2.34; *p* < 0.001), PLR (OR, 1.58; 95% CI, 1.26–1.99; *p* < 0.001), SIRI (OR, 1.68; 95% CI, 1.33–2.11; *p* < 0.001), and AISI (OR, 1.84; 95% CI, 1.45–2.34; *p* < 0.001) were also associated with malignancy. Conversely, higher LMR was inversely associated with malignant histopathology (OR, 0.72; 95% CI, 0.58–0.89; *p* = 0.003).

Multivariable logistic regression models were subsequently constructed to determine whether these associations remained independent of relevant clinical and ultrasonographic characteristics. Because the inflammatory indices are derived from overlapping hematological components and are therefore substantially correlated, they were not entered simultaneously into the same model. Instead, each index was evaluated separately in an otherwise identical multivariable model adjusted for age, sex, serum TSH level, Hashimoto thyroiditis, maximum nodule diameter, composition, echogenicity, margin characteristics, and calcification pattern.

All six inflammatory indices remained independently associated with malignant histopathology after multivariable adjustment. SII retained the strongest positive association with malignancy (adjusted OR, 2.09; 95% CI, 1.60–2.72; *p* < 0.001). NLR also remained independently associated with malignant histopathology (adjusted OR, 1.96; 95% CI, 1.51–2.54; *p* < 0.001). Significant independent associations were similarly observed for PLR (adjusted OR, 1.53; 95% CI, 1.20–1.96; *p* < 0.001), SIRI (adjusted OR, 1.91; 95% CI, 1.47–2.50; *p* < 0.001), and AISI (adjusted OR, 1.96; 95% CI, 1.50–2.54; *p* < 0.001). In contrast, higher LMR remained inversely associated with malignancy (adjusted OR, 0.64; 95% CI, 0.50–0.82; *p* < 0.001). Detailed univariable and multivariable estimates are presented in Table 4.

Assessment of multicollinearity showed low VIF values across all primary multivariable models, with a maximum VIF of 1.41. The VIFs of the individual inflammatory indices ranged from 1.03 to 1.06. In the sensitivity models additionally adjusted for metabolic comorbidities, the maximum VIF was 1.82, indicating no relevant multicollinearity.

In sensitivity analyses additionally adjusting for hypertension, diabetes mellitus, hyperlipidemia, and obesity, the associations between all six systemic inflammatory indices and malignant histopathology remained materially unchanged. SII remained independently associated with malignancy (adjusted OR, 2.10; 95% CI, 1.61–2.75; *p* < 0.001), as did NLR (adjusted OR, 1.96; 95% CI, 1.50–2.55; *p* < 0.001). Similar findings were observed for the remaining inflammatory indices (Supplementary Table S2).

Exploratory analyses using ROC-derived cutoff values yielded findings consistent with the continuous-variable models. SII ≥ 519.7 was associated with increased odds of malignant histopathology (adjusted OR, 3.28; 95% CI, 2.02–5.34; *p* < 0.001), as was NLR ≥1.95 (adjusted OR, 3.10; 95% CI, 1.92–4.99; *p* < 0.001). Significant associations were also observed for the remaining inflammatory indices (Supplementary Table S3).

### 3.6. Incremental Predictive Value of Systemic Inflammatory Indices

To evaluate whether systemic inflammatory indices provided predictive information beyond conventional clinical and ultrasonographic risk factors, each of the six inflammatory indices was separately added to the baseline clinico-ultrasonographic model. The baseline model included age, sex, serum TSH level, Hashimoto thyroiditis, maximum nodule diameter, nodule composition, echogenicity, margin characteristics, and calcification pattern.

The baseline clinico-ultrasonographic model demonstrated an AUC of 0.695 (95% CI, 0.640–0.750). Addition of NLR increased the AUC to 0.751 (95% CI, 0.699–0.803), corresponding to an absolute increase of 0.056. Addition of SII resulted in the highest discrimination, increasing the AUC to 0.761 (95% CI, 0.710–0.811), with an absolute increase of 0.065. The AISI-extended model yielded an AUC of 0.752 (95% CI, 0.701–0.804), corresponding to an absolute increase of 0.057.

Compared with the baseline model, addition of each inflammatory index significantly improved model fit according to likelihood-ratio testing (all *p* < 0.001). The largest numerical increase in AUC was observed with SII (ΔAUC, +0.065), followed by AISI (+0.057), NLR (+0.056), SIRI (+0.052), PLR (+0.034), and LMR (+0.032). For concise presentation, the three indices with the highest individual discriminatory performance are shown in Table 5, whereas incremental model results for all six inflammatory indices are provided in Supplementary Table S1.

An exploratory parsimonious model retaining age, sex, serum TSH, Hashimoto thyroiditis, nodule composition, and margin characteristics showed predictive performance similar to that of the fully adjusted model despite substantially fewer parameters. After addition of SII, the parsimonious model achieved an AUC of 0.754, compared with 0.761 for the full SII-extended model, with corresponding Brier scores of 0.198 and 0.196. The parsimonious SII model also had a lower AIC (428.0 vs. 435.6), and removal of the additional variables was not associated with a significant loss of model fit (likelihood-ratio *p* = 0.790). SII remained independently associated with malignant histopathology in the reduced model (adjusted OR, 2.08; 95% CI, 1.60–2.70; *p* < 0.001). Bootstrap internal validation yielded an optimism-corrected AUC of approximately 0.73 and a calibration slope of approximately 0.85 for the parsimonious SII model, suggesting reduced optimism relative to the fully adjusted model (Supplementary Table S4).

### 3.7. Subgroup Analysis According to Hashimoto Thyroiditis

Of the 352 patients included in the study, 313 (88.9%) did not have Hashimoto thyroiditis and 39 (11.1%) had coexisting Hashimoto thyroiditis. Among patients without Hashimoto thyroiditis, NLR, PLR, SII, SIRI, and AISI were significantly higher in malignant than in benign nodules, whereas LMR was significantly lower. In this subgroup, SII demonstrated the highest discriminatory performance, with an AUC of 0.693, followed by NLR and AISI.

Among patients with Hashimoto thyroiditis, the inflammatory indices showed differences in the same general direction, with higher NLR, PLR, SII, SIRI, and AISI and lower LMR in malignant nodules; however, none of these comparisons reached statistical significance. The discriminatory performance of SII and NLR in this subgroup was modest, with AUCs of 0.661 and 0.642, respectively.

Importantly, formal interaction analyses did not demonstrate significant effect modification by Hashimoto thyroiditis for any of the evaluated inflammatory indices. The interaction *p* values were 0.767 for NLR, 0.579 for PLR, 0.854 for LMR, 0.871 for SII, 0.896 for SIRI, and 0.939 for AISI. No statistically significant interaction between Hashimoto thyroiditis status and any inflammatory index was detected. However, because only 39 patients had Hashimoto thyroiditis, the interaction analyses had limited statistical power, and clinically relevant effect modification cannot be excluded. These findings indicate that the associations between systemic inflammatory indices and malignant histopathology did not differ significantly according to Hashimoto thyroiditis status. Detailed subgroup results are presented in Supplementary Table S5.

### 3.8. Molecular Subgroup Analysis

Definitive molecular testing results were available for 129 patients. Of these, 98 (76.0%) were mutation-negative and 31 (24.0%) harbored at least one detected mutation. Mutation positivity was substantially more frequent among malignant than benign nodules (34.9% vs. 4.3%; *p* < 0.001). The malignancy rate was 93.5% among mutation-positive nodules compared with 55.1% among mutation-negative nodules, corresponding to an odds ratio of 11.81 (95% CI, 2.67–52.27; *p* < 0.001).

Among mutation-positive nodules, BRAF alterations were detected in 17 patients, of whom 16 (94.1%) had malignant histopathology. NRAS mutations were detected in 12 patients, including 11 (91.7%) with malignant histopathology, whereas both KRAS-positive nodules were malignant. Given the small numbers within individual mutation categories, mutation-specific estimates should be interpreted cautiously.

To investigate whether systemic inflammation provided additional information when molecular results were available, incremental modeling was performed within the molecularly characterized subgroup. The clinico-ultrasonographic model demonstrated an AUC of 0.765. Addition of mutation status increased the AUC to 0.824 and significantly improved model fit (likelihood-ratio *p* < 0.001). Mutation positivity remained strongly associated with malignant histopathology after adjustment for clinical and ultrasonographic variables (adjusted OR, 14.35; 95% CI, 2.67–77.01; *p* = 0.002).

In contrast, adding SII to the clinico-ultrasonographic-plus-molecular model did not further improve discrimination or overall model fit. The AUC remained essentially unchanged (0.824 vs. 0.822; ΔAUC, −0.002), and the likelihood-ratio test was not significant (*p* = 0.471). Consistently, SII was not independently associated with malignant histopathology after molecular status was incorporated into the model (adjusted OR, 1.20; 95% CI, 0.73–1.99; *p* = 0.473). In this exploratory molecular subgroup, no statistically significant incremental contribution of SII was detected beyond combined clinico-ultrasonographic and molecular information; however, the limited sample size and model complexity mean that a clinically relevant incremental effect cannot be ruled out. Detailed molecular findings are presented in Table 6, and comparative model performance is provided in Supplementary Table S6.

### 3.9. Internal Validation and Clinical Utility

Bootstrap internal validation demonstrated a reduction in model discrimination after correction for optimism, although the relative performance of the models was preserved. The optimism-corrected AUC was 0.649 for the clinico-ultrasonographic base model, compared with 0.710 for the NLR-extended model, 0.721 for the SII-extended model, and 0.711 for the AISI-extended model. Similarly, the optimism-corrected Brier score was lowest for the SII-extended model (0.215), compared with 0.236 for the base model and 0.218 for both the NLR- and AISI-extended models. Detailed bootstrap validation results are provided in Supplementary Table S7.

**Figure 3:**
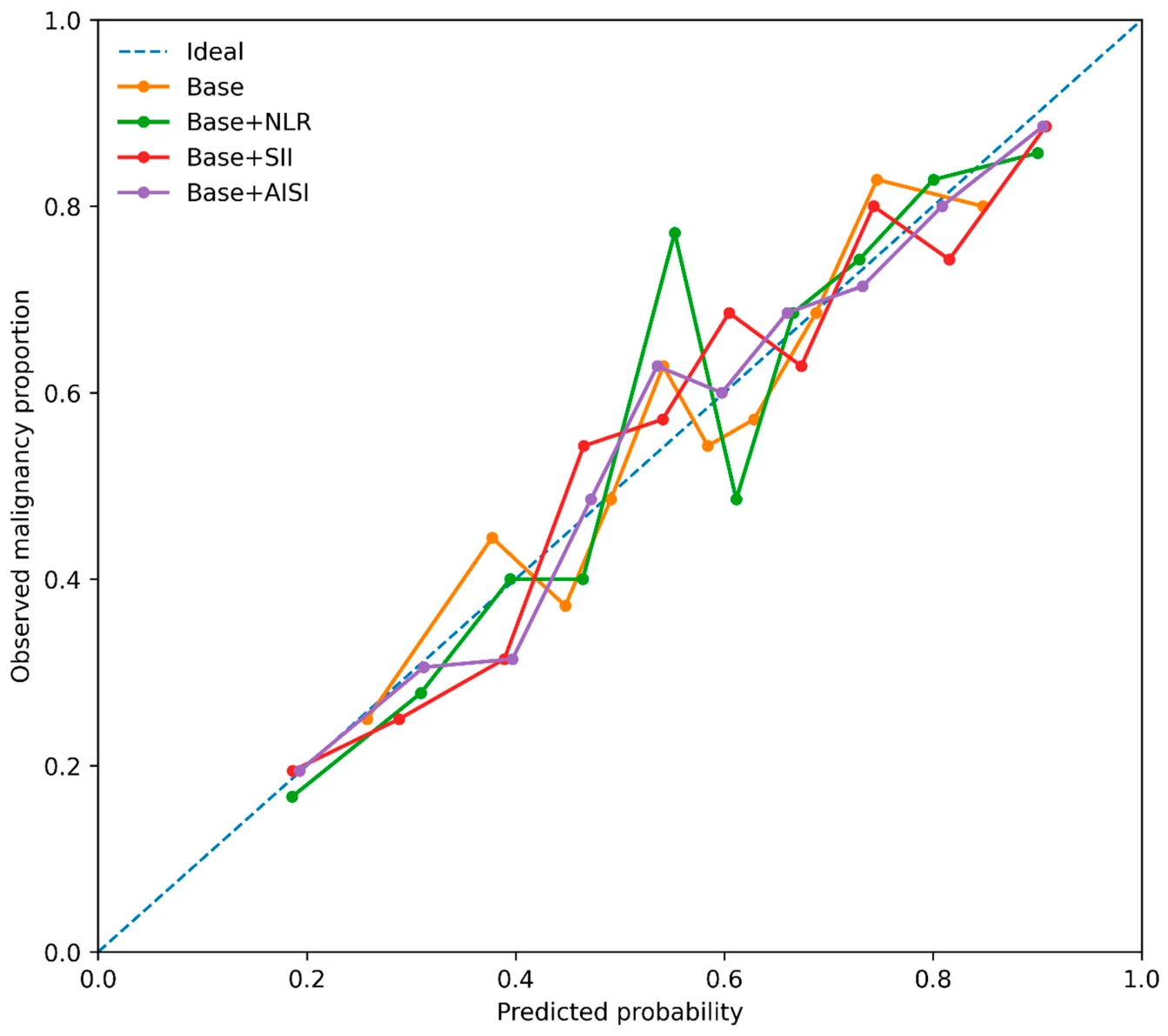
Calibration plots of the clinico-ultrasonographic model and inflammatory-index=extended models for predicting malignant histopathology in Bethesda III thyroid nodules. Calibration curves are shown for the base clinico-ultrasonographic model and the models additionally incorporating NLR, SII, or AISI. The diagonal reference line represents perfect calibration. Internal validation demonstrated optimism-corrected calibration slopes below 1.0 for all models, with the SII-extended model showing the highest corrected slope. Abbreviations: AISI, aggregate index of systemic inflammation; NLR, neutrophil-to-lymphocyte ratio; SII, systemic immune-inflammation index.

Calibration analysis indicated some degree of model overfitting after internal validation. The optimism-corrected calibration slopes were 0.715 for the base model, 0.764 for the NLR-extended model, 0.774 for the SII-extended model, and 0.765 for the AISI-extended model. Although none of the models achieved an ideal calibration slope of 1.0, the optimism-corrected calibration slopes were similar across the extended models, with the SII-extended model showing the numerically highest slope (0.774). Calibration curves are presented in Figure 3.

**Figure 4:**
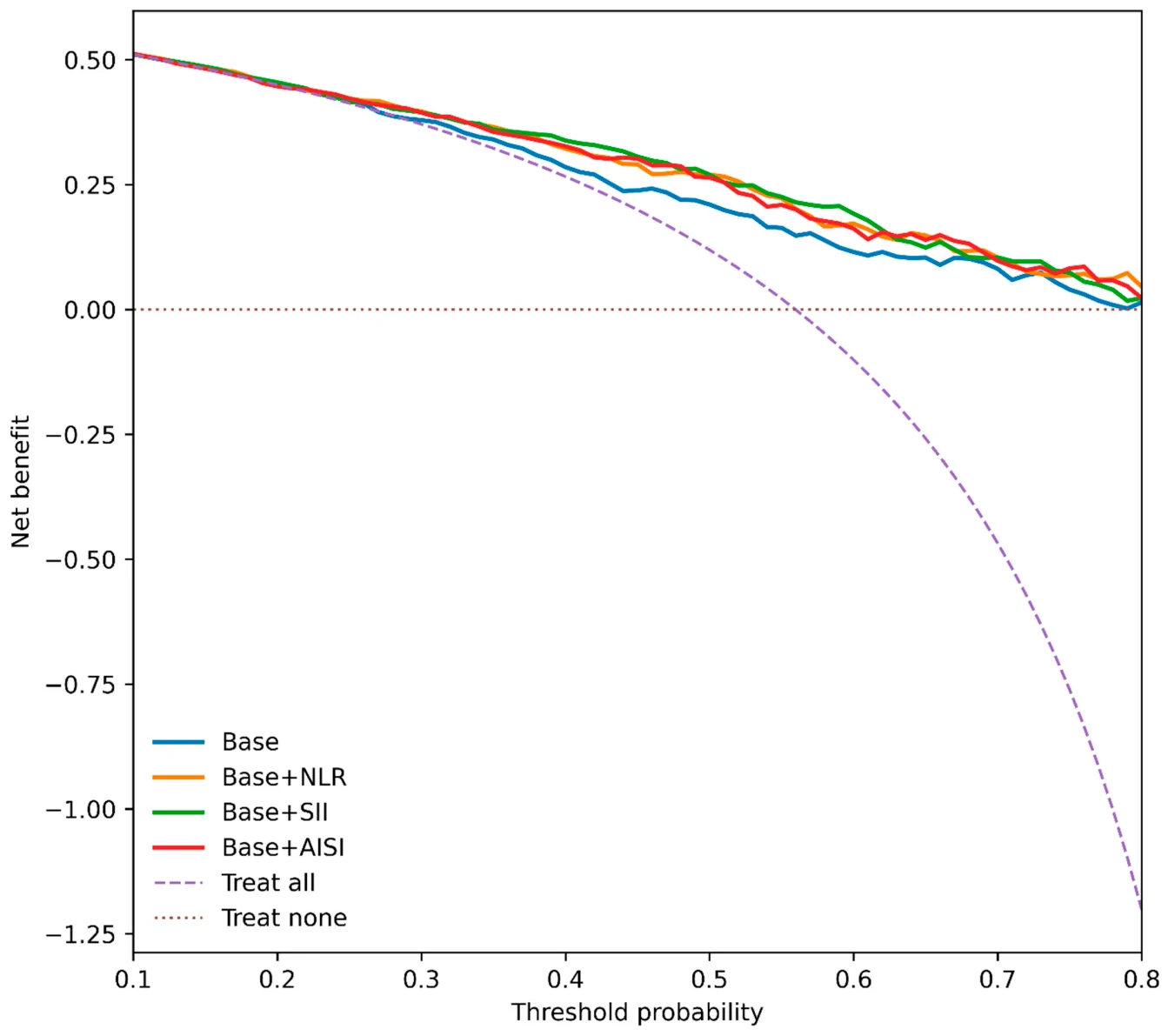
Decision-curve analysis of the clinico-ultrasonographic model and inflammatory-index-extended models for predicting malignant histopathology in Bethesda III thyroid nodules. Decision curves compare the net benefit of the base clinico-ultrasonographic model with models additionally incorporating NLR, SII, or AISI across a range of threshold probabilities. The inflammatory-index-extended models generally provided greater net benefit than the base model across portions of the intermediate threshold range, although the curves were closely overlapping. Abbreviations: AISI, aggregate index of systemic inflammation; NLR, neutrophil-to-lymphocyte ratio; SII, systemic immune-inflammation index.

Decision-curve analysis suggested that the inflammatory-index-extended models provided modestly greater net benefit than the clinico-ultrasonographic model alone across portions of the intermediate threshold range, although the curves were closely overlapping. These findings suggest potential clinical utility, although prospective external validation is required before clinical implementation. Decision curves are shown in Figure 4.

## 4. Discussion

In this retrospective cohort of 352 surgically treated Bethesda category III thyroid nodules, preoperative systemic inflammatory indices were significantly associated with postoperative malignant histopathology. Patients with malignant nodules had higher NLR, PLR, SII, SIRI, and AISI values and lower LMR values than those with benign nodules. All six indices remained independently associated with malignancy after adjustment for relevant clinical and ultrasonographic characteristics. Among the evaluated indices, SII demonstrated the numerically highest discriminatory performance, followed by NLR and AISI, although the discriminatory ability of each index alone remained modest. Importantly, incorporation of SII into the clinico-ultrasonographic model improved discrimination, model fit, and overall predictive performance, and this relative advantage was preserved after bootstrap correction for optimism. By contrast, SII did not provide additional predictive value once molecular mutation status was incorporated into the model. Hashimoto thyroiditis did not significantly modify the associations between the inflammatory indices and malignancy. Collectively, these findings suggest that systemic inflammatory indices, particularly SII, may provide complementary information during the preoperative assessment of Bethesda III nodules, but their clinical value appears to be greatest when interpreted as adjuncts to conventional clinico-ultrasonographic assessment rather than as stand-alone diagnostic tests or substitutes for molecular testing.

The observed associations are biologically plausible within the context of cancer-associated inflammation. Thyroid cancer cells and the surrounding tumor microenvironment can recruit and activate neutrophils, promoting the production of reactive oxygen species and the release of inflammatory, angiogenic, and matrix-remodeling mediators [28]. Lymphocytes, in contrast, have a central role in antitumor immune surveillance, whereas immune escape and suppression of lymphocyte-mediated responses are recognized features of tumor progression [29]. Platelets may facilitate tumor growth, angiogenesis, protection of circulating tumor cells, and metastatic niche formation [30]. Circulating monocytes can differentiate into tumor-associated macrophages; in thyroid cancer, macrophage phenotypes are heterogeneous, with M2-like polarization linked to tumor progression [31,32].

Composite indices such as NLR, SII, SIRI, and AISI integrate several of these circulating cellular components and may therefore capture the balance between systemic inflammatory activation and host immune competence more broadly than individual blood-cell counts [15,33,34,35]. Nevertheless, these indices remain nonspecific and indirect biomarkers and should not be interpreted as direct measurements of the thyroid tumor microenvironment.

Previous studies of systemic inflammatory biomarkers in thyroid cancer have predominantly examined prognostic outcomes or adverse clinicopathological characteristics in patients with established papillary thyroid carcinoma. Higher preoperative NLR and SII have been associated with lymph-node metastasis, whereas higher NLR has also been linked to multifocality, bilaterality, and extrathyroidal invasion in retrospective cohorts [20,21]. However, the available evidence has been heterogeneous across studies and outcomes. A systematic review and meta-analysis including 7599 patients found no significant pooled association of preoperative NLR, PLR, or LMR with disease-free survival in differentiated thyroid cancer [18]. Evidence regarding the use of CBC-derived inflammatory indices to distinguish benign from malignant thyroid nodules is considerably more limited [22].

Studies specifically restricted to Bethesda III/AUS nodules have generally been small and retrospective, with heterogeneous findings. In a surgically treated cohort of 188 AUS/FLUS nodules, Süleyman and Bezirci found higher NLR and SII values in malignant nodules, although only age and absolute neutrophil count remained independent predictors in multivariable analysis [23]. Bostan et al., in a cohort of 200 surgically treated AUS/FLUS patients, reported that higher NLR had only limited predictive value for malignancy, whereas PLR and MPV were not reliable predictors [24]. Similarly, Simsek and Kutluturk found that NLR, PLR, and MPV were not useful markers of malignancy in 62 AUS/FLUS nodules, while microcalcification was the only variable independently associated with malignancy [25].

The present study therefore extends the available literature by evaluating six composite inflammatory indices in a larger and cytologically homogeneous Bethesda III population and by assessing their performance not only through group comparisons and multivariable regression, but also through incremental modeling, calibration, internal validation, decision-curve analysis, autoimmune-thyroiditis interaction testing, and molecular subgroup analysis.

Among the evaluated inflammatory indices, SII demonstrated the numerically highest AUC, followed by NLR and AISI. However, the AUC of 0.687 for SII represents only modest discrimination and is insufficient to support its use as an isolated diagnostic marker. Accordingly, the clinical relevance of SII should not be inferred from its individual discriminatory performance alone. This finding is biologically plausible because SII incorporates neutrophils, lymphocytes, and platelets and may therefore reflect several complementary components of cancer-associated systemic inflammation [33]. Accordingly, the present findings do not support the use of SII, NLR, or any other CBC-derived inflammatory index in isolation to determine whether an AUS nodule should undergo surgery. Their potential value lies instead in their immediate availability, negligible additional cost, and ability to contribute additional risk information when interpreted within a broader clinical framework.

This distinction between association and incremental predictive value is particularly important. Although all six inflammatory indices remained independently associated with malignancy after multivariable adjustment, independent association alone does not establish clinical usefulness. In the present study, the clinico-ultrasonographic model achieved an AUC of 0.695, whereas addition of SII increased the AUC to 0.761. Thus, the potential clinical relevance of SII in this setting lies in its incremental contribution to a multimodal risk-assessment framework rather than in the absolute discriminatory performance of the marker alone. NLR and AISI also improved discrimination, but the SII-extended model showed the largest increase in AUC, the lowest Akaike information criterion, and the lowest Brier score. Bootstrap internal validation reduced the apparent performance of all models, as expected, but the relative ranking was preserved, with the SII-extended model retaining the highest optimism-corrected AUC. These findings suggest that SII provides incremental information beyond conventional clinical and ultrasonographic variables rather than simply reproducing information already captured by established risk factors. Importantly, an exploratory parsimonious model retained similar discriminatory performance with fewer parameters and showed improved optimism-corrected calibration, supporting the robustness of the incremental contribution of SII while reducing model complexity.

However, the clinico-ultrasonographic reference model was constructed from routinely available individual clinical and ultrasonographic variables and did not incorporate standardized Bethesda III cytological subclassification or a formal TI-RADS score. These omissions may have limited the discriminatory performance of the reference model and, consequently, may have increased the apparent magnitude of the incremental improvement associated with SII. Therefore, the observed gain in predictive performance should be interpreted cautiously and requires confirmation against stronger contemporary reference models incorporating standardized cytological subclassification and ultrasound risk-stratification systems.

The calibration and decision-curve findings provide additional context for this incremental improvement. Optimism-corrected calibration slopes below 1.0 indicated some degree of overfitting, underscoring the need for external validation before clinical implementation [36]. Nevertheless, the SII-extended model showed the least evidence of overfitting among the evaluated models. Decision-curve analysis further suggested that models incorporating inflammatory indices provided greater net benefit than the clinico-ultrasonographic model alone across a range of intermediate threshold probabilities, with substantial overlap among the decision curves and only modest numerical differences between the extended models [37].

These findings are encouraging, but they should be interpreted as evidence of potential clinical utility rather than proof of clinical effectiveness. Prospective validation in independent populations is necessary before any inflammatory-index-based model can be incorporated into routine decision-making.

The ultrasonographic findings reinforce the importance of interpreting systemic inflammatory indices as complementary rather than competing markers. Irregular margins were substantially more frequent among malignant nodules, consistent with the established association between suspicious margin characteristics and thyroid malignancy [38,39].

Nodule composition also differed significantly between benign and malignant groups, whereas echogenicity, nodule side, and calcification pattern did not show significant overall differences in the present cohort. The fact that SII improved model performance after adjustment for these ultrasonographic characteristics suggests that systemic inflammation may capture information that is not fully represented by conventional morphologic assessment. At the same time, the modest performance of SII alone emphasizes that it should not replace established ultrasound-based risk stratification.

Hashimoto thyroiditis represents an important potential confounder because autoimmune thyroid inflammation can alter circulating leukocyte profiles and CBC-derived inflammatory indices independently of malignancy [40]. In the present study, Hashimoto thyroiditis was more frequent among patients with benign histopathology, but formal interaction analyses did not demonstrate significant modification of the associations between inflammatory indices and malignancy. Although inflammatory-marker differences appeared more pronounced among patients without Hashimoto thyroiditis, the interaction terms were nonsignificant for all six indices. Although no statistically significant interaction was detected for any of the six inflammatory indices, the Hashimoto thyroiditis subgroup was small, and the interaction analyses were therefore underpowered. Accordingly, the present findings should not be interpreted as evidence that Hashimoto thyroiditis has no modifying effect on the associations between systemic inflammatory indices and malignancy. Larger cohorts with standardized serological, ultrasonographic, and histopathological definitions of autoimmune thyroiditis are required to assess potential effect modification more reliably.

The molecular subgroup findings provide another important perspective. Mutation-positive nodules had a markedly higher malignancy rate than mutation-negative nodules, consistent with the established rule-in value of oncogenic alterations such as BRAF V600E and selected RAS mutations in cytologically indeterminate thyroid nodules [9]. Mutation positivity remained strongly associated with malignancy after adjustment for clinical and ultrasonographic variables, and incorporation of mutation status increased the AUC of the clinico-ultrasonographic model from 0.765 to 0.824. However, more than half of the mutation-negative nodules in this surgically selected cohort were malignant. This high residual malignancy rate should be interpreted in the context of the restricted molecular panel, which assessed a limited set of alterations rather than the broader genomic and gene-expression information incorporated into contemporary multigene classifiers [41,42]. A negative result from such a restricted panel should therefore not be interpreted as equivalent to a negative comprehensive molecular classifier.

Notably, the incremental value of SII was no longer evident once molecular information was incorporated. Addition of SII to the clinico-ultrasonographic-plus-molecular model did not improve discrimination or overall model fit, and SII was no longer independently associated with malignancy in this model. This finding refines the potential clinical role of systemic inflammatory indices. Rather than representing alternatives to molecular testing, inflammatory markers such as SII may be most useful as inexpensive adjuncts when risk assessment relies primarily on clinical and ultrasonographic information or when comprehensive molecular testing is unavailable, inaccessible, or not routinely performed. This interpretation should nevertheless remain cautious because molecular testing was available only in a selected subgroup, was introduced during the later study period, and was not applied systematically across the full cohort.

The biological and pathological heterogeneity inherent to Bethesda III cytology may also explain why no single inflammatory index achieved high diagnostic accuracy. The AUS category encompasses a broad spectrum of cytomorphological abnormalities and is associated with substantial variation in malignancy risk across cytological subgroups and institutions [5,7,43].

Clinical characteristics, ultrasonographic findings, and molecular alterations further refine malignancy risk and management in cytologically indeterminate thyroid nodules [44].

A peripheral blood biomarker reflecting systemic inflammatory status is therefore unlikely to capture the full complexity of this heterogeneous diagnostic category. The present results instead support a multimodal strategy in which systemic inflammatory indices contribute one additional dimension of risk information.

The present study has several strengths. It focused exclusively on Bethesda category III nodules, thereby reducing the heterogeneity introduced by combining different indeterminate cytological categories. All 352 patients had definitive postoperative histopathological diagnoses, providing a uniform reference standard. Six CBC-derived inflammatory indices were calculated from preoperative blood samples, allowing direct comparison of their relative associations and discriminatory performance. The analyses incorporated relevant clinical and ultrasonographic variables, and each inflammatory index was evaluated in a separate adjusted model to minimize the collinearity arising from their overlapping hematological components. In addition, the study extended beyond conventional association analyses by assessing incremental predictive value, calibration, bootstrap-corrected performance, and decision-curve-based net benefit. The availability of Hashimoto thyroiditis and molecular data further allowed two clinically relevant sources of heterogeneity to be examined explicitly.

Several limitations should also be acknowledged. The retrospective, single-center design limits causal interpretation and external generalizability. In addition, the study intentionally included only surgically treated patients, resulting in a malignancy prevalence of 56.0%, which is substantially higher than would be expected in an unselected Bethesda III population. Surgical management was determined through multidisciplinary evaluation and was therefore not random. This may have enriched the cohort for nodules with a higher preoperative probability of malignancy and introduced surgical selection bias. Because corresponding structured clinical and ultrasonographic data and definitive histopathological outcomes were not available for the non-operated group, a reliable direct comparison between surgically and non-surgically managed patients could not be performed. Consequently, the observed malignancy prevalence and model-performance estimates, including discrimination, calibration, and decision-curve findings, should be interpreted as conditional on this surgically selected population and should not be directly extrapolated to unselected Bethesda III cohorts.

In addition, Hashimoto thyroiditis was defined using a composite of serological and ultrasonographic criteria rather than a uniform histopathological definition, and the relatively small Hashimoto subgroup limited the statistical power of the interaction analyses.

Although additional sensitivity analyses accounted for hypertension, diabetes mellitus, hyperlipidemia, and obesity, and patients with active infection or acute inflammatory disease and those receiving systemic corticosteroid or immunosuppressive treatment were excluded, CBC-derived inflammatory indices remain nonspecific. CRP was not routinely available for the entire cohort; therefore, residual confounding from unrecognized or subclinical inflammatory conditions cannot be excluded. Smoking status and continuous BMI values were not consistently available, although obesity and diabetes mellitus were included in sensitivity analyses. Medication histories were reviewed, and patients using drugs likely to affect peripheral blood counts were excluded. Variation within the 30-day preoperative CBC sampling window and unrecognized or asymptomatic SARS-CoV-2 infection may have contributed to residual confounding.

Ultrasonographic characteristics were retrospectively extracted from routine clinical reports and may have been affected by interobserver variability and differences in reporting terminology. In addition, standardized Bethesda III cytological subclassification (nuclear atypia vs. other patterns of atypia) and formal TI-RADS classification were not consistently available throughout the study period. The clinico-ultrasonographic model therefore relied on individual ultrasonographic features rather than on a standardized ultrasound risk-stratification score. These limitations may have reduced the discriminatory performance of the reference model and consequently increased the apparent incremental gain associated with adding SII. Future studies should evaluate systemic inflammatory indices against reference models incorporating standardized Bethesda III subclassification and validated ultrasound risk-stratification systems.

Molecular testing was available only in a selected subgroup and was based on a restricted panel, limiting both statistical power and the generalizability of the molecular analyses. The multivariable molecular subgroup models were based on a relatively small number of patients and should therefore be considered potentially unstable and hypothesis-generating. The optimal inflammatory-index cutoff values were derived and evaluated within the same cohort and should therefore be regarded as exploratory. Although internal bootstrap validation reduced optimism in model-performance estimates, some evidence of overfitting remained, and independent external validation is still required. Finally, the decision-curve findings were derived from the same development cohort and should be interpreted as evidence of potential, rather than established, clinical utility.

## 5. Conclusions

Preoperative systemic inflammatory indices were significantly associated with malignant histopathology in surgically treated Bethesda III thyroid nodules. SII demonstrated the numerically highest individual discriminatory performance and remained independently associated with malignancy after adjustment for clinical and ultrasonographic characteristics. More importantly, addition of SII improved the discrimination and overall performance of a clinico-ultrasonographic model, with this relative advantage persisting after internal bootstrap correction. However, the individual discriminatory performance of SII remained modest, and no significant additional value was detected after molecular mutation status was added, although this exploratory analysis was limited by the small sample size. These findings support the potential role of SII as a low-cost adjunct to conventional preoperative risk stratification, particularly when comprehensive molecular testing is unavailable, while emphasizing that it should not be used as a stand-alone diagnostic tool. Prospective multicenter studies with systematic molecular testing and external model validation are required before clinical implementation.

## Figures and Tables

**Table 1:** Baseline Clinical, Metabolic, and Ultrasonographic Characteristics According to Postoperative Histopathology.

Characteristic	Total (n = 352)	Benign (n = 155)	Malignant (n = 197)	*p* Value
Age, years	51 (42–58)	52 (43–58)	50 (40–59)	0.321
Sex				0.070
Female	280 (79.5)	116 (74.8)	164 (83.2)	
Male	72 (20.5)	39 (25.2)	33 (16.8)	
Hypertension	97 (27.6)	44 (28.4)	53 (26.9)	0.757
Diabetes mellitus	67 (19)	32 (20.6)	35 (17.8)	0.495
Hyperlipidemia	42 (11.9)	18 (11.6)	24 (12.2)	0.870
Obesity	70 (19.9)	35 (22.6)	35 (17.8)	0.261
TSH, mIU/L	1.40 (0.81–2.47)	1.21 (0.79–2.38)	1.60 (0.90–2.56)	0.039
Free thyroxine, ng/dL	1.10 (1.00–1.22)	1.09 (1.00–1.19)	1.10 (1.00–1.24)	0.139
Hashimoto thyroiditis				0.030
Absent	313 (88.9)	131 (84.5)	182 (92.4)	
Present	39 (11.1)	24 (15.5)	15 (7.6)	
Maximum nodule diameter, mm	17.4 (11.5–29.5)	18.4 (12.9–29.2)	17.1 (11.0–29.6)	0.140
Nodule side				0.329
Right	188 (53.4)	76 (49.0)	112 (56.9)	
Left	144 (40.9)	70 (45.2)	74 (37.6)	
Isthmus	20 (5.7)	9 (5.8)	11 (5.6)	
Composition				0.019
Solid	139 (39.5)	53 (34.2)	86 (43.7)	
Predominantly solid	195 (55.4)	89 (57.4)	106 (53.8)	
Predominantly cystic	18 (5.1)	13 (8.4)	5 (2.5)	
Echogenicity				0.235
Isoechoic	223 (63.4)	105 (67.7)	118 (59.9)	
Hypoechoic	36 (10.2)	12 (7.7)	24 (12.2)	
Iso-/hypoechoic	93 (26.4)	38 (24.5)	55 (27.9)	
Margins				<0.001
Smooth	204 (58.0)	109 (70.3)	95 (48.2)	
Irregular	34 (9.7)	6 (3.9)	28 (14.2)	
Ill-defined	114 (32.4)	40 (25.8)	74 (37.6)	
Calcification pattern				0.059
None	276 (78.4)	130 (83.9)	146 (74.1)	
Macrocalcification	46 (13.1)	17 (11.0)	29 (14.7)	
Microcalcification	30 (8.5)	8 (5.2)	22 (11.2)	

Data are presented as median (interquartile range) or n (%). Percentages were calculated using available observations for each variable. Obesity was defined as a body mass index ≥30 kg/m^2^. Continuous variables were compared using the Mann–Whitney *U* test. Categorical variables were compared using Pearson’s chi-square test or Fisher’s exact test, as appropriate. For sex and Hashimoto thyroiditis, *p* values were calculated using the chi-square test with Yates continuity correction.

**Table 2:** Preoperative Systemic Inflammatory Indices According to Postoperative Histopathology.

Index	Benign (n = 155)	Malignant (n = 197)	*p* Value
NLR	1.75 (1.43–2.12)	2.06 (1.68–2.52)	<0.001
PLR	109.8 (89.2–139.8)	126.7 (105.5–156.6)	<0.001
LMR	6.14 (5.20–7.58)	5.80 (4.53–7.22)	0.008
SII	422.3 (320.4–529.3)	547.3 (420.0–692.9)	<0.001
SIRI	0.60 (0.45–0.79)	0.75 (0.56–1.02)	<0.001
AISI	148.4 (106.1–200.0)	203.1 (137.3–291.6)	<0.001

Data are presented as median (interquartile range). Comparisons were performed using the Mann–Whitney *U* test. Abbreviations: NLR, neutrophil-to-lymphocyte ratio; PLR, platelet-to-lymphocyte ratio; LMR, lymphocyte-to-monocyte ratio; SII, systemic immune-inflammation index; SIRI, systemic inflammation response index; AISI, aggregate index of systemic inflammation.

**Table 3:** Diagnostic Performance of Preoperative Systemic Inflammatory Indices for Predicting Malignant Histopathology.

Index	AUC	95% CI	Optimal Cutoff	Sensitivity, %	Specificity, %
SII	0.687	0.631–0.740	519.7	55.8	73.5
NLR	0.661	0.603–0.718	1.95	61.9	65.8
AISI	0.659	0.602–0.714	178.6	61.4	68.4
SIRI	0.634	0.575–0.690	0.83	42.6	80.0
PLR	0.627	0.567–0.685	110.1	72.1	51.6
LMR *	0.582	0.523–0.642	≤4.23	21.3	94.2

Diagnostic performance was assessed using receiver operating characteristic curve analysis. AUC values are presented with bootstrap 95% confidence intervals. Optimal cutoff values were determined using the maximum Youden index and should be considered exploratory because they were derived from the same study cohort. * For LMR, lower values were considered predictive of malignant histopathology. Abbreviations: AISI, aggregate index of systemic inflammation; AUC, area under the receiver operating characteristic curve; CI, confidence interval; LMR, lymphocyte-to-monocyte ratio; NLR, neutrophil-to-lymphocyte ratio; PLR, platelet-to-lymphocyte ratio; SII, systemic immune-inflammation index; SIRI, systemic inflammation response index.

**Table 4:** Univariable and Multivariable Logistic Regression Analyses of Systemic Inflammatory Indices for Predicting Malignant Histopathology.

Index	Univariable OR *	95% CI	*p* Value	Adjusted OR *	95% CI	*p* Value
NLR	1.84	1.45–2.34	<0.001	1.96	1.51–2.54	<0.001
PLR	1.58	1.26–1.99	<0.001	1.53	1.20–1.96	<0.001
LMR	0.72	0.58–0.89	0.003	0.64	0.50–0.82	<0.001
SII	2.09	1.63–2.69	<0.001	2.09	1.60–2.72	<0.001
SIRI	1.68	1.33–2.11	<0.001	1.91	1.47–2.50	<0.001
AISI	1.84	1.45–2.34	<0.001	1.96	1.50–2.54	<0.001

* ORs are expressed per 1-SD increase in the natural-log-transformed inflammatory index and therefore should not be interpreted as the effect of a one-unit increase in the original measurement scale. Each inflammatory index was entered separately into an otherwise identical multivariable model because of substantial overlap among their hematological components. Models were adjusted for age, sex, serum TSH, Hashimoto thyroiditis, maximum nodule diameter, composition, echogenicity, margin characteristics, and calcification pattern.

**Table 5:** Incremental Predictive Value of Systemic Inflammatory Indices Beyond the Clinico-ultrasonographic Model.

Model	AUC (95% CI)	ΔAUC	LR *p* Value	AIC	Brier Score
**Base model**	0.695 (0.640–0.750)	—	—	467.3	0.218
**Base + NLR**	0.751 (0.699–0.803)	0.056	<0.001	440.5	0.199
**Base + SII**	0.761 (0.710–0.811)	0.065	<0.001	435.6	0.196
**Base + AISI**	0.752 (0.701–0.804)	0.057	<0.001	440.8	0.199

The base clinico-ultrasonographic model included age, sex, serum TSH level, Hashimoto thyroiditis, maximum nodule diameter, composition, echogenicity, margin characteristics, and calcification pattern. NLR, SII, and AISI were natural-log transformed and standardized before model entry. ΔAUC represents the absolute change in AUC compared with the base model. Incremental model fit was assessed using the likelihood-ratio test. Lower AIC and Brier score values indicate better model fit and overall predictive performance, respectively. Incremental model results for all six systemic inflammatory indices are provided in Supplementary Table S1. Abbreviations: AIC, Akaike information criterion; AISI, aggregate index of systemic inflammation; AUC, area under the receiver operating characteristic curve; CI, confidence interval; LR, likelihood-ratio; NLR, neutrophil-to-lymphocyte ratio; SII, systemic immune-inflammation index.

**Table 6:** Molecular Test Results According to Postoperative Histopathology in Patients With Definitive Molecular Results.

Molecular Result	Total (n = 129)	Benign (n = 46)	Malignant (n = 83)	*p* Value
**Mutation status**				<0.001
Mutation-negative	98 (76.0)	44 (95.7)	54 (65.1)	
Any mutation-positive	31 (24.0)	2 (4.3)	29 (34.9)	
**Mutation type**				
BRAF-positive	17 (13.2)	1 (2.2)	16 (19.3)	
NRAS-positive	12 (9.3)	1 (2.2)	11 (13.3)	
KRAS-positive	2 (1.6)	0	2 (2.4)	

Data are presented as n (%). Percentages in the benign and malignant columns were calculated within each histopathological group. The association between overall mutation status and malignant histopathology was assessed using Fisher’s exact test. Molecular results reported as equivocal or suspicious rather than definitively positive or negative were excluded from the binary molecular analysis. Abbreviations: BRAF, B-Raf proto-oncogene; KRAS, Kirsten rat sarcoma viral oncogene homolog; NRAS, neuroblastoma RAS viral oncogene homolog.

## Data Availability

Some or all datasets generated during and/or analyzed during the current study are not publicly available because they contain information that could compromise patient confidentiality but are available from the corresponding author on reasonable request.

## Supplementary Materials

The following supporting information can be downloaded at: https://www.mdpi.com/article/10.3390/cancers18193170/s1, Table S1: Incremental Predictive Value of All Systemic Inflammatory Indices Beyond the Clinico-ultrasonographic Model; Table S2: Sensitivity Analysis of the Associations Between Systemic Inflammatory Indices and Malignant Histopathology After Additional Adjustment for Metabolic Comorbidities; Table S3: Exploratory Multivariable Logistic Regression Analyses of ROC-Derived Cutoff Values for Systemic Inflammatory Indices; Table S4: Comparison of Full and Parsimonious Clinico-ultrasonographic Models With and Without SII; Table S5: Association and Diagnostic Performance of Systemic Inflammatory Indices According to Hashimoto Thyroiditis Status; Table S6: Incremental Predictive Value of Molecular Status and SII in the Molecularly Characterized Subgroup; Table S7: Bootstrap Internal Validation of Clinico-ultrasonographic Models With and Without Systemic Inflammatory Indices.
