# Supplementary material for: Preoperative Systemic Inflammatory Indices as Adjunctive Biomarkers for Malignancy Prediction in Bethesda III (AUS) Thyroid Nodules
Source: Cancers (Basel). 2026 Oct 1;18(19):3170. doi: 10.3390/cancers18193170 (PMC13664457; doi:10.3390/cancers18193170)
Supplement: Supplementary file 1 [file cancers-18-03170-s001.zip › cancers-4559350-supplementary.pdf]

Supplementary Materials

Supplementary Table S1. Incremental Predictive Value of All Systemic Inflammatory Indices Beyond the Clinico-ultrasonographic Model.

Model	AUC (95% CI)	ΔAUC	LR *p* value	AIC	Brier score
**Base model**	0.695 (0.640–0.750)	—	—	467.3	0.218
**Base + NLR**	0.751 (0.699–0.803)	+0.056	<0.001	440.5	0.199
**Base + PLR**	0.730 (0.676–0.783)	+0.034	<0.001	457.1	0.208
**Base + LMR**	0.728 (0.675–0.780)	+0.032	<0.001	455.9	0.208
**Base + SII**	0.761 (0.710–0.811)	+0.065	<0.001	435.6	0.196
**Base + SIRI**	0.748 (0.697–0.799)	+0.052	<0.001	443.2	0.201
**Base + AISI**	0.752 (0.701–0.804)	+0.057	<0.001	440.8	0.199

The base clinico-ultrasonographic model included age, sex, serum TSH level, Hashimoto thyroiditis, maximum nodule diameter, composition, echogenicity, margin characteristics, and calcification pattern. Each inflammatory index was natural-log transformed and standardized before being added separately to the base model. ΔAUC represents the absolute change in AUC compared with the base model. Incremental model fit was assessed using the likelihood-ratio test. Lower AIC and Brier score values indicate better model fit and overall predictive performance, respectively. **Abbreviations:** AIC, Akaike information criterion; AISI, aggregate index of systemic inflammation; AUC, area under the receiver operating characteristic curve; LMR, lymphocyte-to-monocyte ratio; LR, likelihood-ratio; NLR, neutrophil-to-lymphocyte ratio; PLR, platelet-to-lymphocyte ratio; SII, systemic immune-inflammation index; SIRI, systemic inflammation response index; TSH, thyroid-stimulating hormone.

Supplementary Table S2. Sensitivity Analysis of the Associations Between Systemic Inflammatory Indices and Malignant Histopathology After Additional Adjustment for Metabolic Comorbidities.

Index	Adjusted OR*	95% CI	*p* value
NLR	1.96	1.50–2.55	<0.001
PLR	1.55	1.20–1.99	<0.001
LMR	0.63	0.49–0.81	<0.001
SII	2.10	1.61–2.75	<0.001
SIRI	1.96	1.50–2.57	<0.001
AISI	2.01	1.54–2.63	<0.001

* ORs are expressed per 1-SD increase in the natural-log-transformed inflammatory index and should not be interpreted as the effect of a one-unit increase in the original measurement scale. Each inflammatory index was entered separately into an otherwise identical multivariable model. Models were adjusted for age, sex, serum TSH, Hashimoto thyroiditis, maximum nodule diameter, composition, echogenicity, margin characteristics, calcification pattern, hypertension, diabetes mellitus, hyperlipidemia, and obesity. The latter four variables were additionally included to assess potential confounding by metabolic conditions associated with chronic low-grade inflammation. **Abbreviations:** AISI, aggregate index of systemic inflammation; CI, confidence interval; LMR, lymphocyte-to-monocyte ratio; NLR, neutrophil-to-lymphocyte ratio; OR, odds ratio; PLR, platelet-to-lymphocyte ratio; SII, systemic immune-inflammation index; SIRI, systemic inflammation response index; TSH, thyroid-stimulating hormone.

Supplementary Table S3. Exploratory Multivariable Logistic Regression Analyses of ROC-Derived Cutoff Values for Systemic Inflammatory Indices.

Index	ROC-derived cutoff*	Adjusted OR	95% CI	*p* value
NLR	≥1.95	3.10	1.92–4.99	<0.001
PLR	≥110.1	2.84	1.75–4.61	<0.001

Index	ROC-derived cutoff*	Adjusted OR	95% CI	*p* value
LMR	≤4.23	6.12	2.64–14.23	<0.001
SII	≥519.7	3.28	2.02–5.34	<0.001
SIRI	≥0.83	3.39	1.97–5.83	<0.001
AISI	≥178.6	3.82	2.34–6.23	<0.001

*Optimal cutoff values were determined using the maximum Youden index. Each inflammatory index was entered separately as a dichotomous variable into an otherwise identical multivariable model adjusted for age, sex, serum TSH, Hashimoto thyroiditis, maximum nodule diameter, composition, echogenicity, margin characteristics, and calcification pattern. For NLR, PLR, SII, SIRI, and AISI, values at or above the indicated cutoff represented the higher-risk category; for LMR, values at or below the cutoff represented the higher-risk category. Because the cutoff values were derived and evaluated within the same cohort, these analyses should be considered exploratory.

Supplementary Table S4. Comparison of Full and Parsimonious Clinico-ultrasonographic Models With and Without SII.

Model	Parameters	AUC	AIC	Brier score	Optimism-corrected AUC	Optimism-corrected Brier score	Optimism-corrected calibration slope
Full base model	13	0.695	467.3	0.218	0.649	0.236	**0.715**
Full model + SII	14	0.761	435.6	0.196	0.721	0.215	**0.774**
Parsimonious base model	8	0.687	460.4	0.220	0.660	0.231	0.805
Parsimonious model + SII	9	0.754	428.0	0.198	0.729	0.211	0.851

The full clinico-ultrasonographic model included age, sex, serum TSH, Hashimoto thyroiditis, maximum nodule diameter, composition, echogenicity, margin characteristics, and calcification pattern. The parsimonious model retained age, sex, serum TSH, Hashimoto thyroiditis, nodule composition, and margin characteristics. SII was natural-log transformed and standardized before model entry. The number of parameters refers to non-intercept model parameters after accounting for indicator coding of categorical variables. Internal validation was performed using 2000 bootstrap resamples. Lower AIC and Brier score values indicate better model fit and overall predictive performance, respectively; a calibration slope closer to 1 indicates better calibration. Abbreviations: AIC, Akaike information criterion; AUC, area under the receiver operating characteristic curve; SII, systemic immune-inflammation index; TSH, thyroid-stimulating hormone.

Supplementary Table S5. Association and Diagnostic Performance of Systemic Inflammatory Indices According to Hashimoto Thyroiditis Status.

Hashimoto status	Index	Benign	Malignant	p value	AUC	Interaction *p**
**Absent**	NLR	1.70 (1.41–2.12)	2.06 (1.68–2.51)	<0.001	0.666	0.767
	PLR	109.4 (88.9–140.9)	126.5 (106.7–156.3)	<0.001	0.628	0.579
	LMR	6.12 (5.20–7.81)	5.82 (4.55–7.14)	0.014	0.581	0.854
	SII	418.1 (320.0–529.3)	542.9 (421.2–688.1)	<0.001	**0.693**	0.871
	SIRI	0.60 (0.45–0.80)	0.75 (0.55–1.01)	<0.001	0.636	0.896
	AISI	147.7 (106.1–196.6)	202.8 (137.4–287.7)	<0.001	0.662	0.939
**Present**	NLR	1.90 (1.68–2.08)	2.02 (1.82–2.95)	0.145	0.642	
	PLR	114.2 (95.8–134.1)	132.6 (95.8–155.0)	0.220	0.619	
	LMR	6.41 (5.20–7.43)	5.40 (4.41–7.48)	0.379	0.586	
	SII	462.3 (339.0–525.5)	589.2 (399.6–830.0)	0.097	**0.661**	
	SIRI	0.63 (0.50–0.75)	0.72 (0.62–1.28)	0.170	0.633	
	AISI	154.3 (107.3–214.1)	221.9 (133.6–346.8)	0.180	0.631	

Data are presented as median (interquartile range). Benign and malignant groups were compared using the Mann–Whitney U test. AUCs were calculated using receiver operating characteristic

analysis; for LMR, lower values were considered predictive of malignancy. * Interaction *p* values were derived from multivariable logistic regression models containing the inflammatory index × Hashimoto thyroiditis interaction term and adjusted for age, sex, serum TSH, maximum nodule diameter, composition, echogenicity, margin characteristics, and calcification pattern. Inflammatory indices were natural-log transformed and standardized before interaction analysis. **Abbreviations:** AISI, aggregate index of systemic inflammation; AUC, area under the receiver operating characteristic curve; LMR, lymphocyte-to-monocyte ratio; NLR, neutrophil-to-lymphocyte ratio; PLR, platelet-to-lymphocyte ratio; SII, systemic immune-inflammation index; SIRI, systemic inflammation response index.

Supplementary Table S6. Incremental Predictive Value of Molecular Status and SII in the Molecularly Characterized Subgroup.

Model	AUC	ΔAUC	LR *p* value	AIC	Brier score
Clinico-ultrasonographic model	0.765	—	—	168.8	0.184
+ Mutation status	**0.824**	**+0.059**	**<0.001**	**155.7**	**0.162**
+ Mutation status + SII	0.822	−0.002	0.471	157.2	0.162

The clinic-ultrasonographic model included age, sex, serum TSH level, Hashimoto thyroiditis, maximum nodule diameter, composition, echogenicity, margin characteristics, and calcification pattern. Mutation status was coded as mutation-positive versus mutation-negative. SII was natural-log transformed and standardized before model entry. ΔAUC represents the absolute change in AUC relative to the immediately preceding model. Incremental model fit was assessed using the likelihood-ratio test. Lower AIC and Brier score values indicate better model fit and overall predictive performance, respectively. **Abbreviations:** AIC, Akaike information criterion; AUC, area under the receiver operating characteristic curve; LR, likelihood-ratio; SII, systemic immune-inflammation index; TSH, thyroid-stimulating hormone.

Supplementary Table S7. Bootstrap Internal Validation of Clinico-ultrasonographic Models With and Without Systemic Inflammatory Indices.

Model	Apparent AUC	Optimism-corrected AUC	Apparent Brier score	Optimism-corrected Brier score	Optimism-corrected calibration slope
Base model	0.695	0.649	0.218	0.236	0.715
Base + NLR	0.751	0.710	0.199	0.218	0.764
**Base + SII**	**0.761**	**0.721**	**0.196**	**0.215**	**0.774**
Base + AISI	0.752	0.711	0.199	0.218	0.765

Internal validation was performed using 2000 bootstrap resamples. Optimism-corrected performance estimates were calculated by accounting for the difference between model performance in each bootstrap sample and its performance when applied to the original dataset. The base clinic-ultrasonographic model included age, sex, serum TSH level, Hashimoto thyroiditis, maximum nodule diameter, composition, echogenicity, margin characteristics, and calcification pattern. NLR, SII, and AISI were natural-log transformed and standardized before model entry. A calibration slope of 1 indicates ideal calibration; values below 1 suggest some degree of model overfitting. **Abbreviations:** AISI, aggregate index of systemic inflammation; AUC, area under the receiver operating characteristic curve; NLR, neutrophil-to-lymphocyte ratio; SII, systemic immune-inflammation index; TSH, thyroid-stimulating hormone.
